# Acute perforation in a gastrojejunocolic fistula after a laparascopic Roux-en-Y gastric bypass: case report

**DOI:** 10.1186/s40792-023-01620-z

**Published:** 2023-03-20

**Authors:** Jos Velleman, Benoit Masereel, Paul Geyskens

**Affiliations:** 1 Department of Plastic, Reconstructive and Aesthetic Surgery, University Hospitals Leuven, Leuven, Belgium; 2 Department of Abdominal Surgery, Sint-Trudo Hospital, 100 Diestersteenweg, 3800 Sint-Truiden, Belgium

**Keywords:** Gastrojejunocolic fistula, Acute perforation, Laparascopic Roux-en-Y gastric bypass

## Abstract

**Background:**

Gastrojejunocolic fistulas are a rare type of fistulas after a laparascopic Roux-en-Y gastric bypass (LRYGB). They are known as a chronic complication. This case report is the first to describe an acute perforation in a gastrojejunocolic fistula after LRYGB.

**Case presentation:**

A 61-year-old woman with a history of a laparascopic gastric bypass was diagnosed with an acute perforation in a gastrojejunocolic fistula. A laparascopic repair was performed by closing the defect in the gastrojejunal anastomosis as well as the defect in the transverse colon. However, 6 weeks later, a dehiscence of the gastrojejunal anastomosis occured. This was reconstructed by an open revision of the gastric pouch and gastrojejunal anastomosis. Long-term follow up showed no recurrence.

**Conclusions:**

Combining the findings of our case with other literature, a laparoscopic repair with wide resection of the fistula, a revision of the gastric pouch and gastrojejunal anastomosis as well as closing the defect in the colon seems to be the best approach in case of an acute perforation in a gastrojejunocolic fistula after LRYGB.

## Introduction

Fistulas after a laparascopic Roux-en-Y gastric bypass (LRYGB) are described in literature. The most common are the gastro-gastric fistulas, between the gastric pouch and the excluded stomach. An analysis of 1292 consecutive patients shows an incidence of 1.2% [1]. However, gastrojejunocolic fistulas are an even more rare type of fistulas after LRYGB [2]. They are described as a chronic complication [2]. To the best of our knowledge, our case report is the first to describe an acute perforation in a gastrojejunocolic fistula after LRYGB.

## Case report

In December 2018, a 61-year-old woman presented herself to the emergency unit with severe epigastric pain since three hours and tachycardia. Physical examination revealed a painfull palpation of the epigastric area and right hypochondrium, as well as a mild rebound tenderness in the epigastric area. A blood test showed a mild raise of the inflammatory markers.

Her current daily medication consisted of pantoprazole 20 mg, paroxetine 20 mg, nortriptyline 50 mg and lormetazepam 4 mg.

She had a medical history of depression, diabetes type II, obestity, osteoporosis, cholecystectomy, tabacco and alcohol abusus. Furthermore, the medical history mentioned a bulbar ulcer. The bulbar ulcer dated from earlier than the digital medical file (earlier than 2002), probably due to NSAID use after a total hip prosthesis. Starting from 2002, there were no signs of an ulcer with a negative endoscopy in november 2011. She underwent a laparascopic short gastric bypass in 2013. During the surgery in 2013, a biliopancreatic limb length of 40 cm and a alimentary limb length of 150 cm was performed. The gastric pouch was constructed with three staplers of 60 mm. The gastro-enterostomy measured 40 mm and was formed by a linear stapler. Chronic diarrhea was present since 2017. In September 2018, gastroscopy and sigmoidoscopy were performed due to melena. Gastroscopy showed an ulcer at the gastrojejunal anastomosis. Sigmoidoscopy was normal. The dose of pantoprazole was increased temporarily to 40 mg twice a day, followed by a maintenance dose of 40 mg a day. In October 2018, the patient was hospitalised at the department of gastroenterology for iron deficiency anemia. A new gastroscopy showed no ulcers or other lesions and a normal anastomosis was visualised. Pantoprazole was decreased to 20 mg a day. One month later, the patient was hospitalised again due to weight loss and hypoalbuminemia with edema. A coloscopy was planned.

Computed tomography at the time of arrival at the emergency unit showed the presence of intra-abdominal free air, including air bubbles around the gastrojejunal anastomosis.

**Fig. 1:**
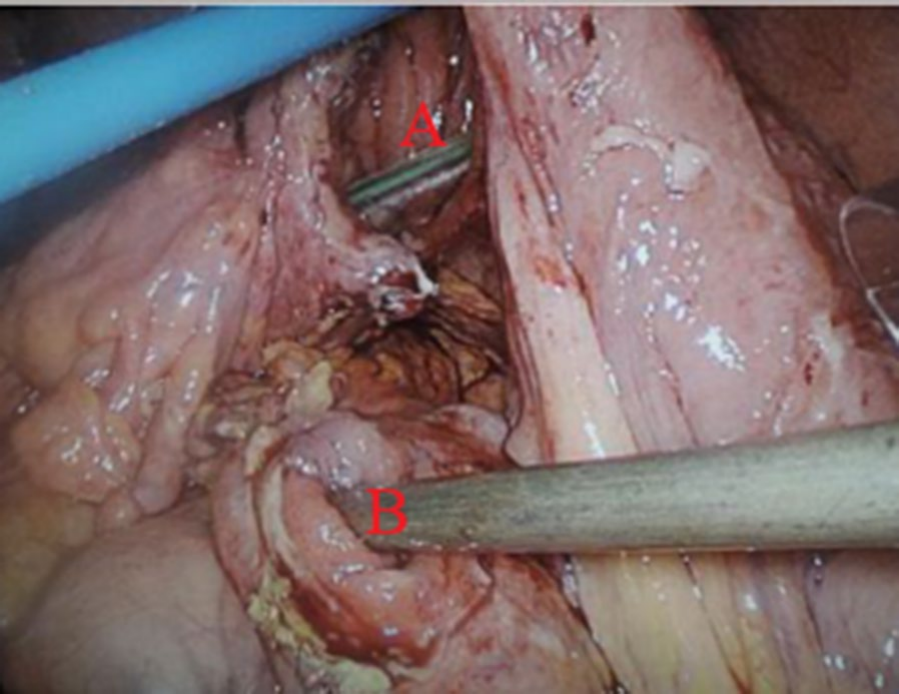
Image of the perforation in the gastrojejunocolic fistula during the laparascopy in December 2018. The defect in the gastrojejunal anastomosis is shown in **A**. The defect in the transverse colon is shown in **B**

**Fig. 2:**
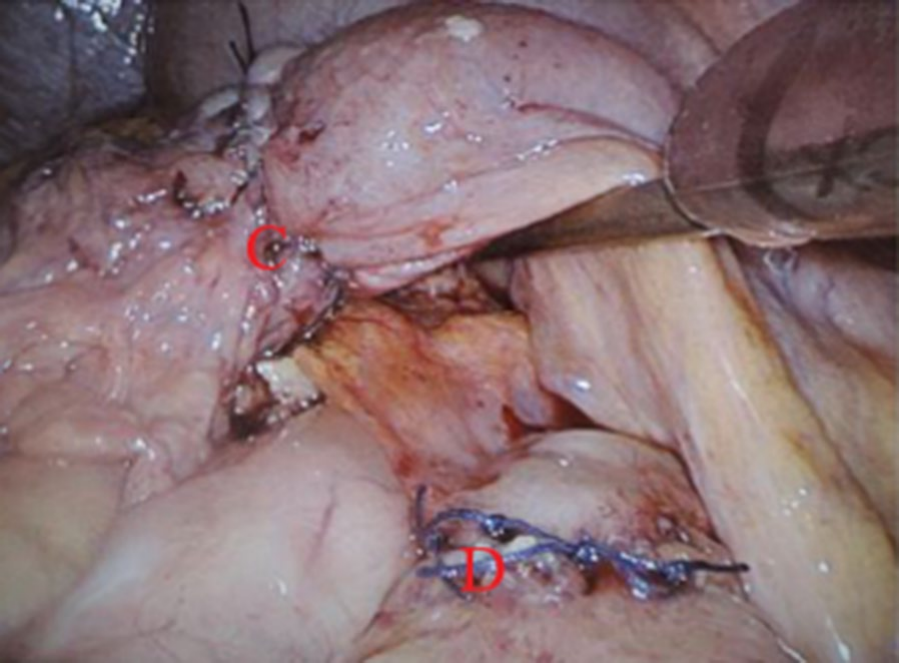
Image during the laparascopy in December 2018 after repairing the defect in the gastrojejunal anastomosis (**C**) and and after repairing the defect in the transverse colon (**D**)

A laparascopic exploration revealed turbid fluid in the upper abdomen. The transverse colon was fixated to the dorsal side of the gastrojejunal anastomosis. Further dissection showed a perforation in a gastrojejunocolic fistula. As a result, a defect of 2.5 cm was found in the transverse colon, as well as in the gastrojejunal anastomosis (Fig. 1). The defect in the transverse colon was repaired by separate stitches using 0 Vicryl. A running suture 2–0 Vicryl was used to repair the defect in the gastrojejunal anastomosis (Fig. 2). A nasogastric tube was left in place. Further inspection of the alimentary limb, biliopancreatic limb, the entero-enterostomy, small intestine common channel and colon was normal. One drain was placed in the pouch of Douglas and one drain was placed under the left hemidiaphragm.

Postoperatively, prophylactic amoxicillin/clavulanic acid was administered intravenously. However, the patient developed a pneumonia, which was treated with intravenous piperacilline/tazobactam. No other postoperative complications occured. The dose of pantoprazole was increased to 40 mg twice a day. Low molecular weight heparines were administered as thrombosis prophylaxis.

However, 6 weeks later, the patient presented herself again to the emergency unit of another hospital with anorexia since 4 days, mild epigastric pain, tachycardia and elevated inflammatory markers. Computed tomography showed free air and fluid in the abdomen and a defect in the gastrojejunal anastomosis.

A laparascopy comfirmed a dehiscence of the gastrojejunal anastomosis, partly covered by the large biliopancreatic stump. There were no visible signs of ischemia at the site of dehiscence. The transverse colon which was repaired in December 2018, was still intact. No other lesions were found. In order to restore the anastomosis, a conversion to a laparotomy was performed. The gastric pouch was stapled with a linear stapler to maintain only vital tissue. The large biliopancreatic stump was shortened by using a linear stapler, followed by an additional running suture 3–0 Monocryl. The mesenteric defect was closed using 0 Ethibond. Subsequently, the gastrojejunal anastomosis was revised by performing a circular stapled gastrojejunal anastomosis, followed by additional separate stitches 3–0 Monocryl. The anvil of the stapler was introduced into the gastric pouch through the opened stump of the alimentary limb. Subsequently, the stump of the alimentary limb was closed by a linear stapler, followed by an additional running suture 3–0 Monocryl. Methylene blue test showed no leakage. At the end of the surgery, the alimentary limb length measured 70 cm. A nasogastric tube was left in place. One drain was placed under the left hemidiaphragm and one drain was placed with the tip nearby the gastrojejunal anastomosis.

Postoperatively, prophylactic piperacilline/tazobactam was administered intravenously during 7 days. Pantoprazole 40 mg twice a day was continued. Low molecular weight heparines were administered as thrombosis prophylaxis. Upper gastrointestinal tract radiography showed a good passage of contrast fluid. There were no signs of leakage. The postoperative stay was free of complications. Pathology report of the biopsies retrieved during this surgery mentioned following results. The gastric pouch showed mucosal and submucosal ischemia and granulation tissue without signs of malignancy. Biopsies of the biliopancreatic limb length and the alimentary limb showed mucosal and submucosal ischemia without signs of malignancy.

The patient was not present at the follow-up consultation abdominal surgery. Nevertheless, during the latest internal medicine consultation in context of osteoporosis in July 2022, no abdominal problems were mentioned by the patient.

## Discussion

To the best of our knowledge, this case report is the first to describe an acute perforation in a gastrojejunocolic fistula after LRYGB. Schulman et al. described a chronic gastrocolic fistula after LRYGB without perforation [3]. A chronic jejunocolic fistula after LRYGB was described by Fronza et al. [4]. Sundeep et al. published a case report of an acute perforation in a gastrojejunocolic fistula [5]. However, this complication occured after a gastroenterostomy performed for recurrent peptic ulcer disease.

Possible symptoms in a gastrojejunocolic fistula are fecal eructation, chronic diarrhea, malnutrition and weight loss [3–5]. In our case, chronic diarrhea, malnutrition and weight loss were reported. However, fecal eructation was not present.

Fistulas after LRYGB are often associated with an ulcer at the gastrojejunal anastomosis or with a chronic leak at the anastomosis [3, 4]. In this case, gastroscopy showed an ulcer at the gastrojejunal anastomosis in September 2018. After increasing the dose of pantoprazole, a new gastroscopy in October 2018 showed no ulcers or other lesions. Reducing the dose of pantoprazole after this negative gastroscopy has possibly contributed to the recurrence of the ulcer with fistula formation.

The cause of an ulceration at the gastrojejunal anastomosis is multifactorial. Diminished perfusion can contribute to the occurrence of ulcers. Moreover, excessive acidity and foreign materials like the suturing material can contibute. Furthermore smoking and NSAIDs show association with ulceration. The role of a Helicobacter pylori infection is controversial. The majority of patients show a response to high doses of proton-pump inhibitors [6–9].

In our case, the initial repair of the acute perforation in the gastrojejunocolic fistula was done laparoscopically by closing the defect in the transverse colon and closing the defect in the gastrojejunal anastomosis. 6 weeks later, an open redo of the gastrojejunal anastomosis was performed in context of a dehiscence of the gastrojejunal anastomosis. Follow-up was 3 years and 6 months.

Concerning chronic fistula, the case presented by Schulman et al. was treated by a laparascopic removal of the fistula and a revision of the gastric pouch and gastrojejunal anastomosis [3]. However, also a simple endoscopic or surgical closure of the chronic fistula is mentioned in literature [3].

Sundeep et al. described the case of an acute perforation in a gastrojejunocolic fistula after a gastroenterostomy performed for recurrent peptic ulcer disease [5]. In this case, an ulcer at the gastro-enterostomy had extended to the transverse colon. The fistula was dissected circumferentially and a wide excision of the ulcer and involved colon was performed during an open revision of the gastrojejunostomy. Bowel continuity was recontructed with a colocolostomy, a Roux-en-Y gastrojejunostomy with feeding jejunostomy [5].

When comparing the report by Sundeep et al. and our case, in both cases, an open surgery with wide excision of the nonvital tissue of the gastric pouch has led to a long term stable result. However, the first repair in our case was possible in a laparoscopic way. Combining these findings, a laparoscopic repair with wide resection of the fistula, a revision of the gastric pouch and gastrojejunal anastomosis as well as closing the defect in the colon seems to be the best approach.

## Conclusion

This case report is the first to describe an acute perforation in a gastrojejunocolic fistula after LRYGB. Chronic diarrhea, malnutrition and weight loss were present. However, fecal eructation, another typical symptom in a gastrojejunocolic fistula, was not present in this patient. An early reduction of the dose of pantoprazole after healing of an ulcer at the gastrojejunal anastomosis has possibly contributed to the recurrence of the ulcer with fistula formation. Combining the findings,of our case with other literature, a laparoscopic repair with wide resection of the fistula, a revision of the gastric pouch and gastrojejunal anastomosis as well as closing the defect in the colon seems to be the best approach in case of an acute perforation in a gastrojejunocolic fistula after LRYGB.

## Data Availability

Provided in the manuscript. If further information is needed, please contact the authors.

## Abbreviation

LRYGB: Laparascopic Roux-en-Y gastric bypass
